# miRNA Expression and Interaction with Genes Involved in Susceptibility to Pristane-Induced Arthritis

**DOI:** 10.1155/2018/1928405

**Published:** 2018-12-16

**Authors:** Jussara Gonçalves Fernandes, Andrea Borrego, José Ricardo Jensen, Wafa Hanna Koury Cabrera, Mara Adriana Correa, Nancy Starobinas, Orlando Garcia Ribeiro, Olga Martinez Ibañez, Marcelo De Franco

**Affiliations:** ^1^Laboratório de Imunogenética, Instituto Butantan, São Paulo 05503000, Brazil; ^2^Seção de Diagnóstico, Instituto Pasteur, São Paulo 01311000, Brazil

## Abstract

Pristane-induced arthritis (PIA) in mice is an experimental model that resembles human rheumatoid arthritis, a chronic autoimmune disease that affects joints and is characterized by synovial inflammation and articular cartilage and bone destruction. AIRmax and AIRmin mouse lines differ in their susceptibility to PIA, and linkage analysis in this model mapped arthritis severity QTLs in chromosomes 5 and 8. miRNAs are a class of small RNA molecules that have been extensively studied in the development of arthritis. We analyzed miRNA and gene expression profiles in peritoneal cells of AIRmax and AIRmin lines, in order to evaluate the genetic architecture in this model. Susceptible AIRmax mice showed higher gene (2025 vs 1043) and miRNA (240 vs 59) modulation than resistant AIRmin mice at the onset of disease symptoms. miR-132-3p/212-3p, miR-106-5p, miR-27b-3p, and miR-25-3p were among the miRNAs with the highest expression in susceptible animals, showing a negative correlation with the expression of predicted target genes (*Il10*, *Cd69*, and *Sp1r1*). Our study showed that global gene and miRNA expression profiles in peritoneal cells of susceptible AIRmax and resistant AIRmin lines during pristane-induced arthritis are distinct, evidencing interesting targets for further validation.

## 1. Introduction

Rheumatoid arthritis (RA) is a chronic autoimmune disease that affects joints and causes persistent synovial inflammation and articular cartilage bone destruction [1]. It affects about 1% of the population, with higher prevalence in women. Disease etiology is unknown, but influenced by both genetic and environmental factors [2, 3]. Experimental models of arthritis are widely used to study the mechanisms of autoimmunity and inflammation and also to search for new therapeutic targets. Pristane-induced arthritis (PIA) in mice resembles rheumatoid arthritis in its histopathological and immunological features, mainly in polymorphonuclear infiltration, pannus formation, cartilage and bone destruction, rheumatoid factor and anticollagen type II antibody production, and chronic development of the disease [4].

Mouse lines genetically selected to maximal (AIRmax) or minimal (AIRmin) acute inflammatory response induced by s.c. injection of polyacrylamide beads [5, 6] also differ widely in their susceptibility/resistance to PIA [7]. These mice have been extensively used to study the genetic susceptibility to various experimental disease models and the acute inflammatory response [8–10]. In the PIA model, genome mapping detected significant QTLs for PIA severity in chromosomes 5 (*Prtia2*) and 8 (*Prtia3*) and suggestive QTLs in chromosomes 7, 17, and 19 [11].

Several studies have demonstrated the involvement of small RNAs, known as miRNAs in the development of RA. miRNAs are a class of small, noncoding, RNA molecules with approximately 21 nucleotides in length that can regulate gene expression by reducing the ability of specific mRNAs to direct the synthesis of their encoded proteins [12]. They likely participate in most developmental and physiologic processes, with involvement in, but not limited to, cell proliferation and differentiation, regulation of lipid metabolism, and modulation of insulin secretion. The importance of miRNA-mediated regulation of gene expression for the prevention of autoimmunity and maintenance of normal immune system functions has been described [13]. Studies in humans have detected altered miRNA expression in RA patients when compared to controls or osteoarthritis patients [14–16].

miRNAs can be detected in body fluids without invasive procedures and thus may be used as prognostic or diagnostic biomarkers for specific conditions, such as rheumatic diseases [17].

In this work, we investigated the differences in miRNA expression by peritoneal cells of pristane-treated AIRmax and AIRmin mice and their involvement in the expression of their potential target genes by microarrays. Numerous evidences have shown that miRNA targets are affected at the mRNA level and, therefore, comparison of microRNA and mRNA expression data is useful in identifying and evaluating the impact of gene dysregulation in the PIA model.

## 2. Materials and Methods

### 2.1. Animals

Male and female AIRmax and AIRmin mice (two to four months old) bred and maintained in the animal facility of Immunogenetics Laboratory in Butantan Institute, São Paulo, Brazil, were used in the experiments. All procedures were approved by the Institutional Animal Care and Use Committee of the Butantan Institute and University of São Paulo (098/13 CEAU).

### 2.2. Pristane-Induced Arthritis (PIA)

Mice were injected twice i.p. with 0.5 ml of the nonimmunogenic mineral oil pristane (2,6,10,14-tetramethylpentadecane, Sigma Chemical Co., San Diego, USA) at a 60-day interval and were examined twice weekly for arthritis development up to 170 days. Arthritis incidence was assessed, and severity was recorded for each paw according to the following scoring scheme: 0—no signs of arthritis, 1—mild swelling of the toes or ankle joint, 2—moderate swelling, and 3—severe swelling and/or ankylosis. The maximum score possible for any animal was 12. Phenotypes were assessed by two independent observers, and the animals were considered arthritic when the mean score assigned by the two observers was ≥2 [18]. Mice were euthanized at 120 and 170 days after pristane or saline injection in order to obtain peritoneal cells.

### 2.3. Peritoneal Cells

The peritoneal cavity was washed with 5 ml of ice-cold RPMI 1640 medium. The cell suspension was centrifuged 3 times to remove excess oil and resuspended in complete RPMI medium. Viable cells were counted in a hemocytometer chamber by Trypan blue exclusion.

### 2.4. Total RNA Extraction

Total RNA was isolated from approximately 1 × 10^7^ peritoneal cells with the mirVana™ miRNA isolation kit (Ambion, Austin, TX) according to the manufacturer's protocol. RNA degradation was assessed using the Agilent 2100 Bioanalyzer (Agilent Technologies, Santa Clara, CA, USA). The minimum value for RNA integrity number (RIN) used was 8.7. cDNA from miRNA was synthesized using TaqMan™ MicroRNA Reverse Transcription Kit (Applied Biosystems, Poland) according to the manufacturer's protocol.

### 2.5. miRNA Real-Time PCR

The expressions of mmu-miR-146a-5p, mmu-miR-155-5p, mmu-miR-132-3p, mmu-miR-130b-3p, mmu-miR-106-5p, and mmu-miR-27b-3p were determined using a TaqMan™ miRNA assay (Applied Biosystems) according to the manufacturer's protocol. miRNA expression was normalized against the geometrical mean of RNA202 and U6 small nuclear RNAs. The Ct values were calculated and determined by the 2^−∆∆Ct^ method [19, 20].

### 2.6. Global Gene and miRNA Expression

The reagents used for microarrays were from “GeneChip” Affymetrix mouse 2.0 ST bioarrays—35 k genes for mRNAs and “Gene Chip” miRNA 4.0 Array for miRNAs (Affymetrix Inc., Santa Clara, CA, USA), following the manufacturer's protocols. Briefly, 1 *μ*g of total RNA was transcribed to cDNA by *in vitro* transcription with the primers T7-(N)6 oligo(d) and complementary double-stranded cRNA and subsequently labeled with biotin. Fragmented cRNA samples were prepared for hybridization on GeneChip Probe Arrays (Affymetrix, USA) and incubated for 17 h. Then, the chips were washed and stained with streptavidin Cy5 and scanned in a Fluidics Station 450 (Affymetrix). Signals were detected and evaluated using GeneChip Operating Software (GCOS). Spots below the detection level of the negative controls were excluded, as well as spots with irregular shapes or intensities close to background levels.

### 2.7. Microarray Data Analysis

Criteria for defining values to identify differentially expressed genes (DEGs) were based on statistical analysis using unpaired one-way ANOVA within the Transcriptome Analysis Console 3.0 (TAC) software, provided by Affymetrix, with FDR < 0.05 (false discovery rate). This program performs statistical analysis to obtain a list of differentially expressed genes. To increase the power of the statistical analysis, we only considered the genes and miRNAs whose differences in expression between controls and experimental animals were at least 3 or 2, respectively.

### 2.8. miRNA-RNA Interaction

Selection of mRNA targets for the differentially expressed miRNA (miRDEGs) was carried out in the miRSystem database (miRNA Integration System for Target Gene Prediction—http://mirsystem.cgm.ntu.edu.tw/). miRSystem is a database which integrates seven well-known miRNA target gene prediction softwares: DIANA, miRanda, miRBridge, PicTar, PITA, rna22, and TargetScan [21]. For our analysis, we considered the interactions predicted at least in three of these softwares. Cytoscape (http://www.cytoscape.org/) was used to display the predicted miRNA-mRNA interactions.

### 2.9. Statistical Analyses

The significance of differences between mean values was calculated using one-way ANOVA followed by Tukey's multiple comparison tests, *p* < 0.05 (^∗^), using Prism 5.0 software (GraphPad, USA).

## 3. Results

### 3.1. mRNA Expression Profile in Peritoneal Cells

**Figure 1:**
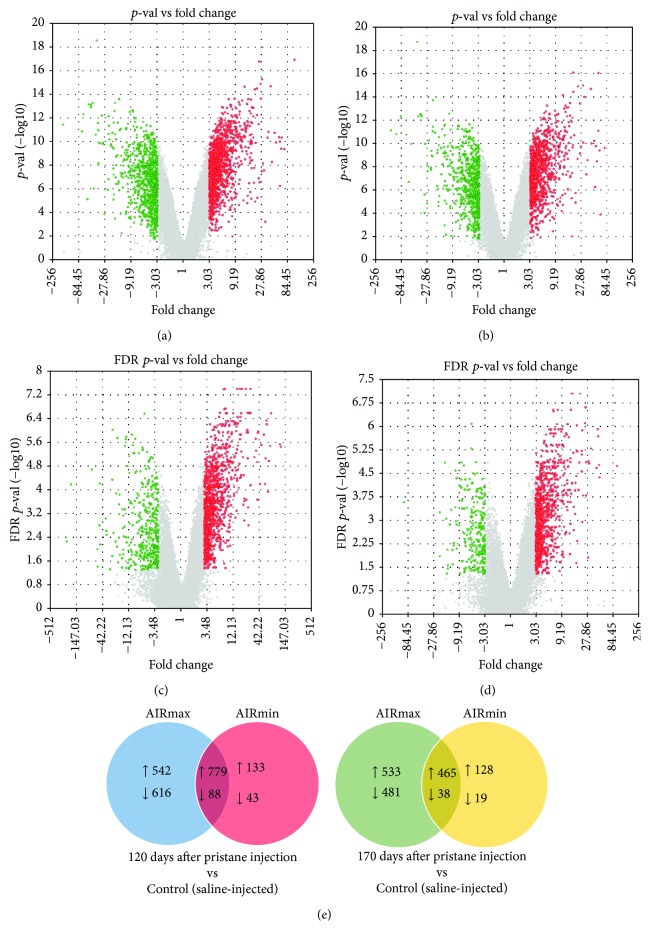
Gene expression modulation in AIRmax and AIRmin mice after pristane injection. (a–d) Upregulated (red) and downregulated (green) genes 120 and 170 days after pristane injection in peritoneum. (a) AIRmax 120 days, (b) AIRmax 170 days, (c) AIRmin 120 days, (d) AIRmin 170 days, and (e) exclusive and common DEGs in AIRmax and AIRmin lines injected with pristane and compared to controls. Differentially expressed genes (DEGs) were detected using unpaired one-way ANOVA in the Transcriptome Analysis Console 3.0 (TAC) software, with FDR < 0.05 considering a minimum difference of 3 times for differentially expressed genes. *N* = 5 animals per group.

We compared mRNA expression from pristane-injected and control mice. Gene modulation was observed in both lines, at 120 and 170 days after pristane injection, and arthritic AIRmax mice had 1321 upregulated and 704 downregulated genes compared to controls after 120 days (Figure 1(a)). AIRmin animals (with no signs of disease) had 912 upregulated and 131 downregulated genes (Figure 1(c)). 542 genes were exclusively upregulated, and 616 were exclusively downregulated in AIRmax mice, while in AIRmin mice 133 genes were exclusively upregulated and 43 downregulated (Figure 1(e)). At 170 days, AIRmax animals had 998 upregulated and 519 downregulated genes (Figure 1(b)), whereas AIRmin mice had 593 upregulated and 57 downregulated genes (Figure 1(d)). 533 genes were exclusively upregulated in AIRmax mice and 128 were exclusives in AIRmin mice. Yet, 481 genes were exclusively downregulated in AIRmax mice and only 19 in AIRmin mice (Figure 1(e)). Some genes that were modulated in AIRmax animals, with reported involvement in human rheumatoid arthritis and in animal models, are shown in Table 1.

### 3.2. Differentially Expressed Genes in Chromosomes 5 and 8

**Figure 2:**
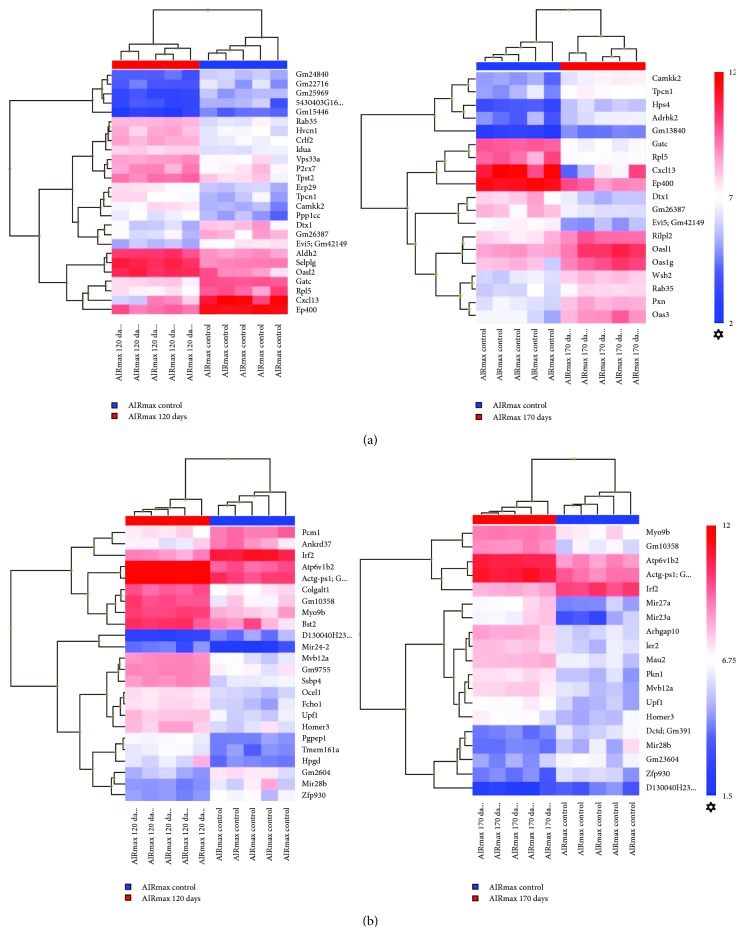
Up- and downregulated genes in AIRmax lines, located in the 1-LOD score confidence interval (CI) of *Prtia2* (Chr 5) and *Prtia3* QTL (chr 8) (>3-fold). (a) Chromosome 5: left—120 days; right—170 days. (b) Chromosome 8: left—120 days; right—170 days. Differentially expressed genes (DEGs) were detected using unpaired one-way ANOVA, with FDR < 0.05.

We evaluated the differentially expressed genes on chromosomes 5 and 8, where significant QTLs for severity to PIA were detected [11]. In AIRmax mice, 150 genes were modulated in chromosome 5 in both periods and 112 genes were modulated in chromosome 8. Twenty-six genes at 120 days and 19 genes at 170 days were situated in the *Prtia2* locus (Chr 5) 1-LOD score confidence interval (CI), while 24 genes at 120 days and 19 at 170 days were situated in the *Prtia3* locus (Chr 8) 1-LOD score CI (Figure 2).

### 3.3. miRNA Expression Profile in Peritoneal Cells

**Figure 3:**
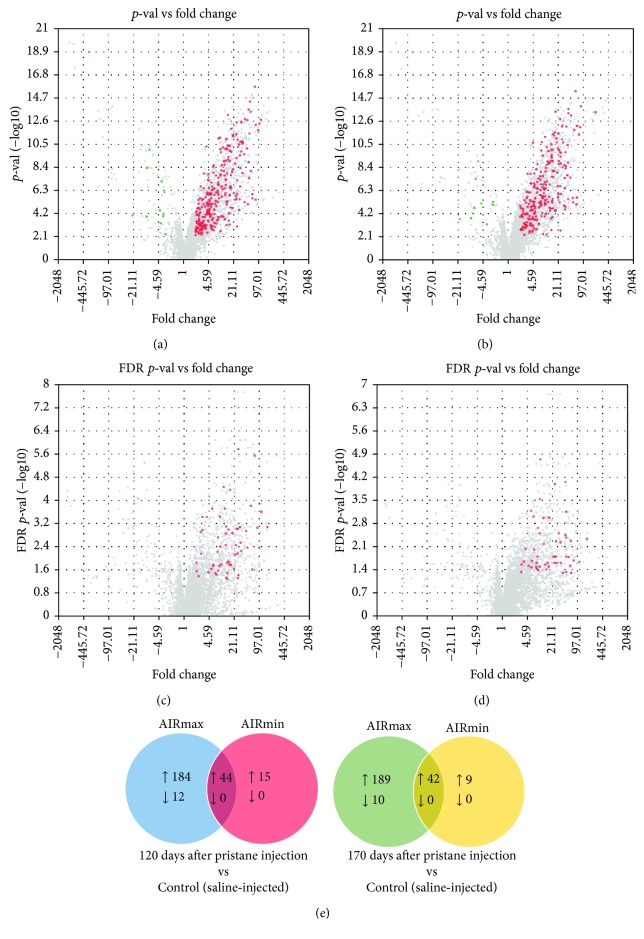
Upregulated (red) and downregulated (green) miRNAs after pristane injection in peritoneum. (a) AIRmax 120 days, (b) AIRmax 170 days, (c) AIRmin 120 days, (d) AIRmin 170 days, and (e) exclusives and commons miRDEGs in AIRmax and AIRmin lines injected with pristane and compared to controls. Differentially expressed miRNAs (miRDEGs) were detected using unpaired one-way ANOVA in the Transcriptome Analysis Console 3.0 (TAC) software, with FDR < 0.05 considering a minimum difference of 2 times for differentially expressed genes. *N* = 5 animals per group.

Divergent miRNA expression profiles were observed in AIRmax and AIRmin lines after pristane injection, when compared to their respective controls. At 120 days, 228 miRNAs were upregulated in AIRmax mice, in which 184 were upregulated exclusively in this line (Figures 3(a) and 3(e)). 59 miRNAs were upregulated in AIRmin mice, with 15 exclusives to this line (Figures 3(c) and 3(e)). Twelve miRNAs were downregulated in AIRmax mice while no miRNA was downregulated in AIRmin mice. At 170 days, 233 miRNAs were upregulated in AIRmax mice (189 exclusively) and 10 miRNAs were exclusively downregulated (Figures 3(b) and 3(e)). AIRmin mice had 51 upregulated miRNAs (9 exclusively) while no miRNA was downregulated (Figures 3(d) and 3(e)).

### 3.4. Target Prediction and Integration with Genome-Wide mRNA Expression

**Figure 4:**
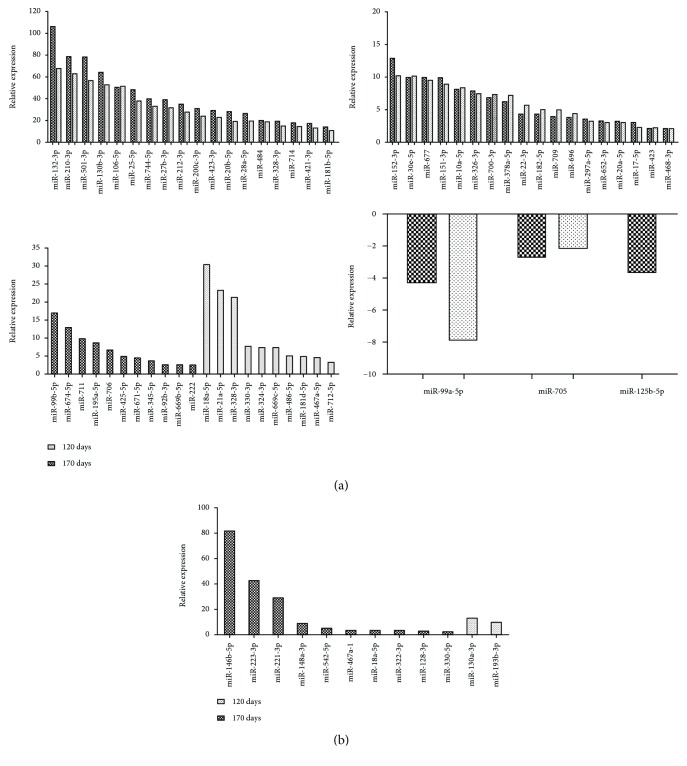
(a) Up- and downregulated miRNAs after pristane injection. Only the miRNAs predicted to interact with their targets in at least 3 different databases were shown. (a) miRNAs exclusively modulated in AIRmax mice and (b) miRNAs exclusively modulated in AIRmin mice. Differentially expressed miRNAs (miRDEGs) were detected using unpaired one-way ANOVA, with FDR < 0.05 and considering a minimum 2-fold difference for miRDEGs.

We selected specific upregulated miRNAs from each group for subsequent miRNA-mRNA interaction networks. To analyze the predicted target mRNAs for each miRDEG, we used the target prediction tool miRSystem database that contains validated data on interaction between miRNA and target genes from TarBase and miRecords [21]. In general, regulation by a given miRNA in a specific tissue can be confirmed only for a fraction of predicted targets. Therefore, in order to increase the likelihood of predicting genuine miRNA targets, only the interactions predicted by at least 3 different algorithms were taken into account for analysis among miRDEGs. In this way, 57 miRNAs were selected among the exclusively upregulated miRNAs in the AIRmax line (Figure 4). Among miRNAs exclusively downregulated in AIRmax mice, 3 were selected as shown in Figure 4(a). AIRmin animals had 12 exclusively upregulated miRNAs (Figure 4(b)), and no miRNA was downregulated.

### 3.5. miRNA Validation with RT-PCR

The genes selected for real-time PCR studies were based on microarray data to confirm the data observed after 120 and 170 days. In contrast to microarray analysis, miRNA-146a-5p and miR-130b-3p were upregulated in AIRmax and AIRmin mice after pristane injection and miR-146a-5p was more expressed in AIRmax controls in comparison to AIRmin control mice. The miRNAs 132-3p, 106-5p, and 27b-3p were upregulated in AIRmax animals but not in AIRmin animals, in agreement with microarray data. Additionally, these miRNAs had higher expression in AIRmax than in AIRmin mice 120 days after pristane injection. After 170 days, the expression of these miRNAs did not differ among these lines. miRNA-155-5p was not modulated after pristane injection.

### 3.6. miRNA-RNA Interaction Network

**Figure 5:**
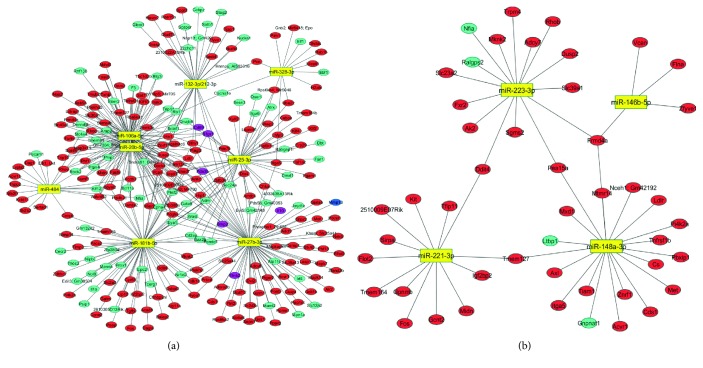
mRNA-miRNA interaction network. (a) miRNAs upregulated in AIRmax mice and their interaction with predicted target genes; (b) miRNAs upregulated in AIRmin mice and their interaction with predicted target genes. Red and blue = upregulated genes; green and purple = downregulated genes. The interaction network was built with Cytoscape 3.4.0.

Due to the complexity of the miRNA-mRNA interaction network, we selected miRNAs with highly significant difference in expression between experimental and control mice at 120 and 170 days. The upregulated miRNAs found in AIRmax mice regulate a higher number of targets than in AIRmin (Figure 5). However, most of the target genes were upregulated in both lines. An example of the inverse correlation among miRNA and mRNA expressions could be observed for the *Bmp2* gene in AIRmax mice. miR-27b-3p and miR-106a-5p have this gene as a predicted target. The BMP-2 protein belongs to the TGF-*β* family and is involved in the differentiation of mesenchymal cells into osteoblasts [22]. *Il10* and *Efnb2* genes were also shown to be regulated by miR-27b-3p. *S1pr1* and *Cd69* genes were downregulated in AIRmax mice and were predicted targets of miR-106a-5p, miR-25-3p, and miR-20b-5p. Several further inverse correlations between mRNA and miRNA expression could be observed in this network (Figure 5).

## 4. Discussion

In the present work, we used two mouse lines to investigate the involvement of peritoneal cell miRNAs in susceptibility or resistance to experimental arthritis using genome-wide microarrays. AIRmax and AIRmin lines represent an interesting model for studying the mechanisms involved with susceptibility to arthritis, as shown by the detection of several QTLs modulating the intensity of the nonspecific inflammatory component of the disease in these lines [11].

The peritoneal cell gene and miRNA expression profiling in both lines after pristane injection identified exclusively over- and underexpressed genes and miRNAs. Overall, pristane induced the modulation of several genes in AIRmax and AIRmin lines in both time points analyzed. This modulation was higher in AIRmax mice. These animals had about 2-fold more modulated genes than the AIRmin line (2025 vs 1043). This difference reflects mainly the number of downregulated genes, which was about 5-fold higher in AIRmax animals (704 vs 131). In previous microarray analyses using the paws of these animals, the AIRmax line also showed about 5-fold more downregulated genes than AIRmin and 2-fold more upregulated genes [11]. The same gene expression profile was also observed in the subcutaneous tissue of these lines after Biogel injection [23]. Although different tissues and stimuli have been analyzed, these results indicate that the selective pressure during phenotypic selection acted in general inflammatory regulation mechanisms.

The identification of DEGs among these lines is also important for correlating with QTLs (quantitative trait loci) previously mapped by De Franco et al. [11] on chromosomes 5 (*Prtia2*) and 8 (*Prtia3*). Several DEGs were clustered in the confidence interval (CI) of these QTLs (Figure 2), being possibly related to the regulation of pristane-induced arthritis.

One of the AIRmax-upregulated genes located in the CI of the chromosome 5 QTL is *P2rx7*, a candidate for susceptibility to autoimmune inflammatory diseases (Table 1). P2RX7 is a cellular receptor expressed in hematopoietic cells such as macrophages, microglia, and some lymphocyte subsets [24] and is activated by high extracellular ATP concentrations, which act as a potent DAMP (damage-associated molecular pattern) when released by damaged tissue. For this reason, P2RX7 is considered an innate immune system danger sensor [25]. Activation of P2RX7 leads to formation of the NALP3 inflammasome, caspase-1 activation, and the consequent release of IL-1*β* and IL-18, while prolonged P2RX7 activation leads to apoptosis [26].

Elliott et al. [27] indicated *P2rx7* for the human *SLEB4* and murine lupus *lbw3* susceptibility loci. The underlying hypothesis is that P2RX7-induced programmed cell death might be a source of autoantigens or represent a catastrophic cell death that destroys the ability of the host to remove such material. In pristane-induced lupus models, pristane was able to induce apoptosis in rat peritoneal and lymph node cells [28, 29].

Vorraro et al. [9] mapped a QTL in chromosome 7 regulating the number of infiltrating cells in 24-hour Biogel-induced exudates and ex vivo IL-1*β* production following LPS and ATP stimulation. As mentioned above, ATP is required for P2RX7 activation and rapid IL-1*β* release. In addition, AIRmax and AIRmin animals are polymorphic for this gene. Together, these data show that *P2rx7* is a strong candidate on chromosome 5 for the susceptibility to PIA. The differential expression and clustering of genes located in this QTL reinforce the significance of the genetic factors of this chromosomal region in the modulation of pristane-induced arthritis.

AIRmax animals injected with pristane also upregulated the complement C5 receptor genes *C5aR1* and *C5aR2* (Table 1). C5aR has been associated with collagen-induced arthritis (CIA). These genes were upregulated in peripheral blood and synovial macrophages and neutrophils of arthritic patients and in mice injected with anticollagen antibodies, and their expression in the patients' joints was detected mainly in macrophages [30]. Knockout of these receptors conferred protection from the disease in mice [30, 31]. Ribeiro and coworkers [32] detected high concentrations of C5a fragment in the Biogel-induced inflammatory exudate of AIRmax animals when compared to AIRmin. Consistent with these results, AIRmax sublines homozygous for the *Slc11a1* gene R allele, which lack C5a, are more resistant to arthritis than the homozygous mice for the *S* alleles [33]. Expression of these genes in the PIA-resistant AIRmin line was unaffected by pristane.

miRNA expressions after pristane injection were also distinct in AIRmax and AIRmin mice. At 120 days, 184 miRNAs were upregulated and 12 downregulated exclusively in AIRmax animals. That regulation was similar (189 upregulated and 12 downregulated) at 170 days. In contrast, the AIRmin line upregulated 15 and 10 miRNAs at 120 and 170 days, respectively; no downregulated miRNA was detected. The higher number of downregulated genes observed in AIRmax mice may be a consequence of the upregulation of miRNAs in their peritoneal cells.

Fifty-seven miRNAs, highly expressed in AIRmax animals, were selected using online miRSystem software [21] (Figure 4), based on the criteria described in Materials and Methods. That approach increased the likelihood of selecting true miRNA-RNA interactions, since not all algorithms yield the same results. It is important to emphasize that, since the main objective of this work was to evaluate the differences between susceptible and resistant lines that may lead to the definition of their phenotype, we restricted our analyses to those genes and miRNAs that were only up- or downregulated exclusively in one of the lines—and whose expressions were more significantly modified in treated animals than in controls.

Most of the up- or downregulated miRNAs have not been ascribed roles in experimental or human arthritis development. Instead, many of those miRNAs are described in terms of their roles in suppressing or inducing several types of malignant tumors, although many have been shown to be involved in the regulation of important biological processes in the development of autoimmune diseases such as inflammation. We therefore sought to identify important pathways in which those miRNAs participated and which could explain their modulation in our model—eventually leading to the identification of new arthritis-related miRNAs in experimentally induced arthritis.

miR-132-3p was the most upregulated miRNA in the susceptible mouse line in microarrays and qRT-PCR (106- and 7-fold higher at 120 days and 67- and 4.5-fold higher at 170 days, respectively). Expression of that miRNA has been found to increase in the peripheral blood mononuclear cells (PBMCs) of rheumatoid arthritis patients [14]. In that study, one of the patients with the active disease showed unaltered levels of that and other miRNAs related to disease after two months of treatment with methotrexate. Those results indicate that the high expressions of miRNAs in that patient were related to unresponsiveness to the treatment. miR-132-3p may therefore play a key role in systemic conditions related to joint inflammation, which would explain its high expression in the peritoneum of susceptible AIRmax animals.

**Figure 6:**
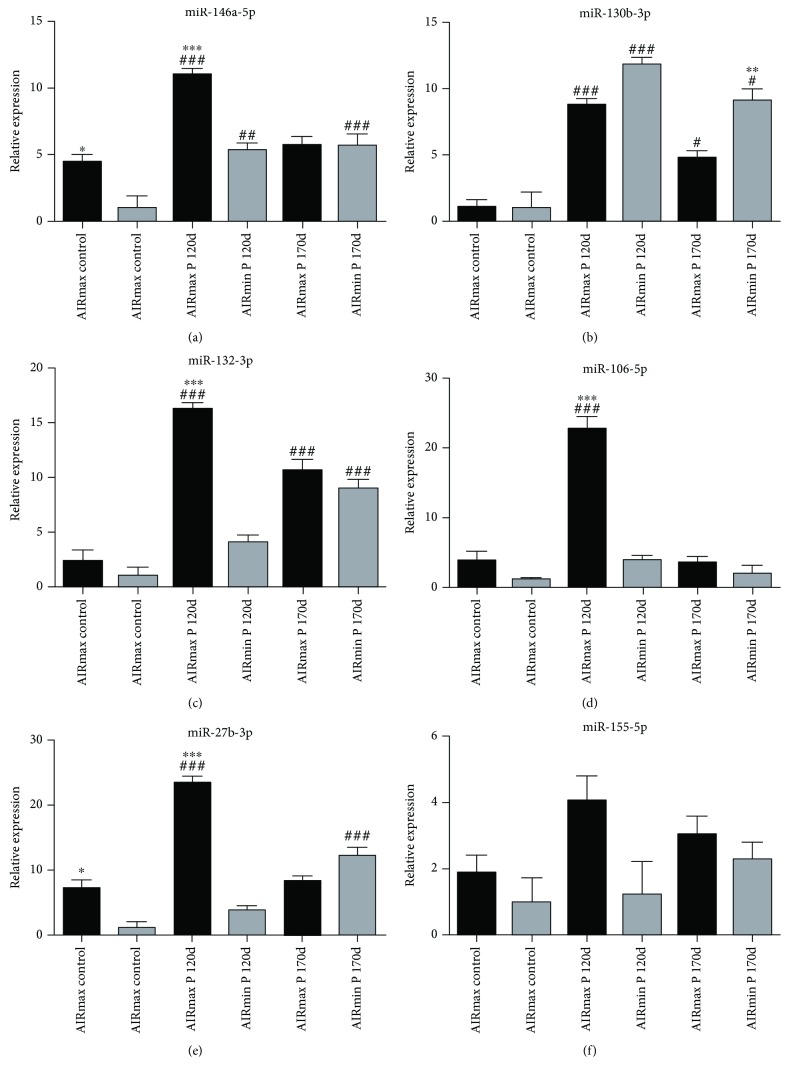
Relative gene expression determined by qRT-PCR in peritoneal cells of AIRmax and AIRmin mice 120 and 170 days after pristane injection (*n* = 5 mice/group). Data expressed as mean ± standard error of two independent experiments; statistical analysis by ANOVA followed by Tukey's post hoc tests. ^∗^*p* < 0.05 between AIRmax and AIRmin; ^#^*p* < 0.05 between control and pristane-injected animals.

miR-132-3p and miR-212-3p are members of the same family (located on chromosome 11 in mice) that forms the miR-212/132 cluster, and they have similar seed sequences. That cluster, induced by the activation of AhR in inflammatory bowel disease, was able to promote an inflammatory response by inducing the Th17 response and suppressing IL-10 production [34]. The Il10 gene was downregulated in peritoneal cells in AIRmax mice (Table 1), indicating that there may be an indirect regulation of the expression of that cytokine by those miRNAs. IL-10 is an important anti-inflammatory cytokine that inhibits proinflammatory mediator production and lymphocyte proliferation, thus playing a protective role in autoimmune diseases. IL-10 has been shown to contribute to the prevention of arthritic inflammation in macrophages during collagen-induced arthritis development [35]. That gene can be regulated by different miRNAs, including miR-27b-3p, which is highly upregulated in that line (Figures 4, 5, and 6).

Cd69 and S1pr1 (specifically targeted by 106a-5p, 25-3p, and 20b-5p miRNAs) were downregulated in AIRmax mice (Table 1 and Figure 5). CD69 is a leukocyte receptor induced in lymphocytes and macrophages after activation. Sancho et al. [36] demonstrated that CD69^−/−^ and CD69^+/−^ mice had an exacerbated form of collagen-induced arthritis (CIA) when compared to controls and that CD69 was capable of inducing TGF-*β*2 synthesis. TGF-*β*2 is an anti-inflammatory cytokine, and null mutations in that gene can lead to severe inflammatory disorders; that gene regulates the production of inflammatory cytokines and has protective effects in the CIA model [36, 37]. Tgfb2 was the most downregulated gene in the AIRmax line (40-fold), while CD69 was approximately 6-fold downregulated. The S1pr1 gene, on the other hand, has been shown to be important for the differentiation of osteoblasts [22]. The inhibition of osteoblast differentiation contributes to bone loss in RA as well as a decreased ability of those lesions to heal [38].

The expressions of miR-181b-5p and IL-6 were shown to be inversely correlated following stimulation with LPS, and IL-6 was shown to be a direct target of miR-181b-5p [39], demonstrating the critical role of the posttranscriptional control of IL-6 by miR-181b-5p in endotoxin tolerance. The expressions of miR-181b-5p and IL-6 were also inversely correlated in susceptible AIRmax mice (Table 1 and Figure 4). Although IL-6 did not appear as a target for miR-181b-5p in our interaction network (which considered at least 3 different algorithms), the data from the TargetScan database (which is widely used in the literature to predict miRNA-RNA interactions) indicated that gene as a possible target of miR-181b-5p. An important role of IL-6 has been reported in the in vitro inhibition of osteoclast progenitors mediated by the disruption of RANK signaling [40]. Osteoclasts are required for articular bone resorption and are responsible for bone erosion in RA [38, 41]. The unbalanced expression of the genes that promote osteoclastogenesis and inhibit osteoblast differentiation may represent a mechanism stimulating bone erosion and increasing disease severity in AIRmax animals. Histological analyses of the paws of the AIRmaxSS subline did, in fact, show bone loss in addition to the destruction of cartilage [42].

Soto et al. [43] compared the gene expression profiles of the rat collagen-induced arthritis model (CIA) with human RA (using paw and knee synovial tissue, respectively). Comparing the DEGs in our model with the model used by Soto et al., we observed that two genes upregulated in AIRmax mice (*Mmp13* and *Gpsm3*) were also upregulated in CIA rats (Table 1).

The *MMP13* and *GPSM3* genes play significant roles in rheumatoid arthritis in humans, and the *GPSM3* gene has been associated with the risk of developing autoimmune diseases [44]. Polymorphisms associated with decreased transcription have been inversely correlated with the risk of developing arthritis. The reduced expression of GPSM3 was observed to prevent neutrophil migration mediated by LTB4 (leukotriene B4) and CXCL8 to arthritic joints [44]. Additionally, mice deficient for the *Gpsm3* gene were protected from arthritis induced by anticollagen antibodies that reduced CCL2 and CX3CL1-mediated migration of myeloid cells [45]. GPSM3 acts as a direct NLRP3 inhibitor, reducing IL-1-beta but not TNF-alpha secretions in the peritoneum [46]. It was also demonstrated that GPSM3 is required for a proper chemokine signal in leukocytes, interfering in their functions during inflammation events [47]. These fine regulated mechanisms are altered in the arthritis onset and progression. Those genes are located on chromosomes 17 and 7 in mice, where suggestive QTLs for experimental arthritis were detected in our model [11]. The miRanda database identified Gpsm3 as a predicted target of miRNA-151-5p, which is downregulated in AIRmax mice. Since that interaction was only predicted in that database, it was not considered in our results, although the high expression of *Gpsm3* as a consequence of the downregulation of miRNA-151-3p should not be completely ruled out.

MMP-13 (or collagenase-3) hydrolyzes type 2 collagen and may favor the destruction of cartilage in arthritic joints. In rheumatoid arthritis, IL-1*β* and TNF-*α* produced by macrophages in the connective tissue stimulate the production of that MMP by articular chondrocytes [48]. Additionally, a key role has been attributed to some genetic loci encoding metalloproteinases in bone destruction [1]. The expression of that MMP increased 10-fold in AIRmax mice (Table 1), but remained unaltered in pristine-treated AIRmin animals. Vonk and coworkers [49] looked for different miRNAs expressed in healthy and osteoarthritis (OA) patients and found that miRNA-148a levels in healthy subjects were approximately 10-fold higher than those seen in patients with the disease. Transfection of miR-148a-3p into cells of OA patients resulted in decreased MMP-13 expression (which had increased in those patients), suggesting that the miRNA (which was also 8-fold more expressed in the resistant AIRmin line) (Figures 4(b) and 5(b)) may play a protective role in that disease by regulating MMP-13 expression induced by pristane, with consequent cartilage destruction.

In a second analysis, Soto et al. [43] identified 30 genes differentially expressed in RA as compared to controls. Of those 30 genes,*Pde3b*, *Tgfb2*, and *Fam120c* were downregulated in both RA patients and AIRmax mice; *Tgfb2* showed a significant protective effect in arthritis models as discussed above.

Many miRNAs are over- or underexpressed in autoimmune diseases such as SLE [50, 51] and rheumatoid arthritis (RA) [52], and different investigators have reported that miR-146a is altered in those diseases [17]. That microRNA was upregulated in both AIRmax and AIRmin lines in both qRT-PCR (Figure 6) and microarray analyses. Interestingly, that miRNA showed a higher expression in AIRmax than in AIRmin control mice 120 days after pristane injection. Increased expression of miRNA-146a has been well-documented in the PBMCs of patients with arthritis. That microRNA has two known targets: Traf6 (TNF receptor-associated factor 6) and Irak1 (interleukin-1 receptor-associated kinase 1), which both stimulate TNF-*α* production [53]. The expressions of those molecules were not altered in those patients, suggesting that in spite of increased miRNA-146a levels, it was unable to regulate TRAF6/IRAK. Therefore, it is not known exactly how the high expression of that miRNA is related to the increased levels of TNF-*α* in RA [52]. Traf6 and Irak1 expressions were unchanged in AIRmax and AIRmin mice.

Despite its high incidence and severe phenotype, RA still has no cure in spite of many efforts to produce effective therapy treatments. Further studies should therefore be carried out to better understand the functions and mechanisms of miRNAs in the immune system and in arthritis development. The AIRmax and AIRmin lines constitute interesting tools for mapping inflammatory disease-modifying genes and miRNAs, in addition to being a valid animal model for the human disease in respect to similar gene pathways and miRNAs. Our study demonstrated that those lines have distinct gene and miRNA expression profiles, which may be partly responsible for their different phenotypes.

## 5. Conclusions

Our study showed that the distinct peritoneal cell gene and miRNA expression profiles of AIRmax and AIRmin lines are correlated with their divergent PIA susceptibility phenotypes, evidencing interesting targets for further validation.

## Figures and Tables

**Table 1:** Genes involved with human RA and experimental arthritis models, up- and downregulated in AIRmax mice after pristane injection (*n* = 5 per group). Significant fold change (FC) was calculated using one-way ANOVA, with FDR < 0.05.

Genes	AIRmax pristane 120 days/control (FC)	FDR	AIRmax pristane 170 days/control (FC)	FDR
MMP13	10.4	0.001	10.32	0.000587
C5AR1	3.36	0.002	—	—
P2RX7	3.25	0.009	—	—
GPSM3	3.2	0.0003	—	—
C5AR2	3.19	0.004	3.41	0.015568
S1PR1	−3.42	0.000778	−3.63	0.036394
IL-10	−3.93	0.002	—	—
BMP2	−4.31	0.001	−5.11	0.000521
CD69	−6.04	0.002	—	—
EFNB2	−10.24	0.000114	−13.98	0.000003
IL-6	−24.6	0.0002	−15.45	0.003381
TGFB2	−40.36	2.05*E* − 07	−44.4	7.58*E* − 07

## Data Availability

The data files used to support the findings of this study are available from the corresponding author upon request.
